# Assessment of serum bile acid profiles as biomarkers of liver injury and liver disease in humans

**DOI:** 10.1371/journal.pone.0193824

**Published:** 2018-03-07

**Authors:** Lina Luo, Jiri Aubrecht, Dingzhou Li, Roscoe L. Warner, Kent J. Johnson, Julia Kenny, Jennifer L. Colangelo

**Affiliations:** 1 Pharmacokinetics, Dynamics & Metabolism, Medicine Design, Pfizer Inc., Groton, Connecticut, United States of America; 2 Drug Safety Research & Development, Pfizer Inc., Groton, Connecticut, United States of America; 3 University of Michigan Medical School, Ann Arbor, Michigan, United States of America; 4 University of Prince Edward Island, Charlottetown, Prince Edward Island, Canada; University of Basque Country, SPAIN

## Abstract

To assess the potential of individual bile acids (IBA) and their profiles as mechanistic biomarkers of liver injury for humans in real world situations, we interrogated samples collected under minimum controlled conditions (ie subjects were not fasted). Total bile acids (TBA) have been considered to be biomarkers of liver injury for decades, and more recently, monitoring of IBA has been proposed for differentiation of variety of etiologies of liver injury. We established a LC-MS/MS methodology to analyze nine IBA, generated reference ranges, and examined effects of age, gender, and ethnicity for each IBA. Furthermore, we evaluated the ability of IBA and their profiles to detect hepatic injury in subjects with a broad range of liver impairments. To date, our study utilized the largest total cohort of samples (N = 645) that were divided into 2 groups, healthy or liver impaired, to evaluate IBA as biomarkers. The TBA serum levels in the Asian ethnic group trended higher when compared to other ethnic groups, and the serum concentrations of IBA, such as glycocholic acid (GCA), glycochenodeoxycholic acid (GCDCA), chenodeoxycholic acid (CDCA), and taurochenoxycholic acid (TCDCA) were significantly increased. To our knowledge, this report is the first to describe ethnic differences in serum concentrations of IBAs. In patients with hepatic impairments, with the exception of deoxycholic acid (DCA), the concentrations of IBAs were significantly elevated when compared with healthy subjects. The conjugated bile acids displayed greater differences between healthy subjects and subjects with hepatic impairments than non-conjugated bile acids. Furthermore, the subjects with hepatic impairments exhibited distinct profiles (signatures) of IBAs that clustered subjects according the nature of their liver impairments. Although additional studies are needed, our data suggested that the analysis of IBA has the potential to become useful for differentiation of various forms of liver injury.

## Introduction

Better biomarkers of liver injury are needed to aid in the early detection of liver diseases, as well as facilitate the development of safer drugs. Over thirty million Americans (one in ten) have liver disease, which includes over 100 different forms of the disease (www.liverfoundation.org). This includes thirty thousand individuals diagnosed with primary liver cancer, one of the few cancers with increasing incidence, as well as the millions diagnosed with hepatitis B and C. Furthermore, drug induced liver injury is the most frequent cause of market withdrawal for drugs [1]. The development of new biomarkers is a challenging and lengthy process requiring extensive data sets that demonstrate their diagnostic utility. It requires validated analytical assays and thorough evaluation of biomarker performance according to its context of use in large populations.

The measurement of total bile acids (TBA) is considered a biomarker of liver function and an indicator of hepatobiliary impairment or disease. TBA measurement corresponds to the sum of more than 20 individual bile acids (IBA) that are synthetized by the liver, modified by gut bacteria, and involved in complex enterohepatic circulation. Primary bile acids, cholic acid (CA) and chenodeoxycholic acid (CDCA), in addition to downstream secondary bile acids generated by intestinal microflora, make up the majority of the bile acid pool in humans [2]. The bile acid conjugation process results in less toxic and more water-soluble bile acid species that are capable of protecting against damage from toxic hydrophobic bile acids, which can cause increased oxidative stress and cell death signaling [3, 4]. Altered serum bile acid profiles have been reported in rodents with bile duct hyperplasia and ligation [5, 6], also for many chronic liver diseases including cholestasis, primary biliary cirrhosis and nonalcoholic fatty liver diseases [7–9]. Furthermore, changes in bile acid composition can potentiate hepatotoxicity through pro-inflammatory mechanisms, membrane damage, or cytotoxicity [6, 10, 11]. Therefore, there is considerable interest in IBA as potential biomarkers of liver damage and disease [12–14]. The fact that individual bile acids have been identified in multiple studies indicates that they are potentially valuable as biomarkers.

In our study we evaluated the potential of IBA as mechanistic biomarkers of liver injury in humans, as applicable in real world situations. Serum samples were collected under minimum controlled conditions (*ie* subjects were not fasted) and analyzed with a validated LC-MS/MS method for nine bile acids. Reference ranges were established for each IBA, and the effects of age, gender and ethnicity on IBA concentrations were evaluated. In addition, we assessed the potential of IBA profiles (signatures) to differentiate individual liver impairments. Our study comprised the largest number of samples to date (N = 645) and complemented recent publications on bile acids in healthy humans. To our knowledge, this study was the first to evaluate the effect of ethnicity on IBA concentrations.

## Materials and methods

### Chemicals and reagents

CA was purchased from Alfa Aesar (Ward Hill, MA), taurocholic acid (TCA) from Acros Organics (Pittsburgh, PA) and glycocholic acid (GCA) from Pfaltz & Bauer (West Chester, PA). CDCA, glycochenodeoxycholic acid (GCDCA), taurochenoxycholic acid (TCDCA), deoxycholic acid (DCA), glycodeoxycholic acid (GDCA), taurodeoxycholic acid (TDCA) and deoxy-2,2,4,4-d4 acid (DCA-d4) were purchased from Sigma-Aldrich (St. Louis, MO). Cholic-2,2,4,4-d4 acid (CA-d_4_), glycocholic-2,2,4,5-d4 acid (GCA-d_4_), chenodeoxycholic-2,2,4,4-d4 acid (CDCA-d4) and glycochenodeoxycholic-2,2,4,4-d4 acid (-GCDCA-d4) were purchased from C/D/N ISOTOPES Inc. (Quebec, Canada). Taurocholic 2,2,3,4,4-d_5_ acid (TCA- d_5_) and taurochenodeoxycholic-2,2,3,4,4-d_5_ acid (TCDCA-d_5_) were purchased from Toronto Research Chemicals Inc. (Ontario, Canada). HPLC grade methanol, acetonitrile, water and formic acid were purchased from Honeywell Burdick & Jackson (Muskegon, MI). Charcoal stripped serum was purchased from Bioreclamation (Westbury, NY).

### Sample collection from human subjects

The study was conducted in the principles of the Declaration of Helsinki and was approved by the local independent ethics committee. Samples were collected from the Pfizer Clinical Research Unit (CRU), Pfizer Worldwide Research & Development in Groton, and the University of Michigan health care system (UM) under an approved IRB (2000–005). This study was specifically approved and performed in accordance to the UMHS/Medical School Institutional Review Board (IRBMED) guidelines and regulations. All subjects (or guardians if minors) gave written informed consent for their serum to be used in research.

#### Healthy volunteers/subjects

Samples were defined as healthy based upon normal chemistry ranges (ALT, AST, ALP, TBILI, glucose, blood urea nitrogen, serum creatinine and creatine kinase) and no significant clinical diagnosis (S3 Table). Subjects whose values for one or more of the above tests exceeded 2-fold increase of the normal reference range were not classified as healthy. In addition, any healthy subject who had an ongoing health problem or immunological flare was omitted from the cohort. Most samples were collected from subjects who were attending routine health examinations and the fasting status for the majority of these was not documented.

#### Hepatic injury subjects

Samples were collected from subjects with Clinically Significant Diseases at the time of admission to the UM Hospital which indicated impaired liver function, and whom had at one point, elevated AST and ALT values greater than two times normal levels (ALT>70 U/L; AST>60 U/L). Hepatic injury subjects were grouped by their primary diagnosis type, including, Liver Transplant, Hepatic Carcinoma, Coronary Artery Disease (CAD), Coronary Heart Disease (CHD), Cirrhosis and Liver Injury (hepatitis B or C, ethanol cirrhosis, drug abuse, or transaminitis/hepatic congestion), Pulmonary Diseases and Acetaminophen (APAP) Toxicity (S4 Table).

Serum samples were recovered from serum-separator tubes following centrifugation of whole blood at 3000 x g for 10 min at room temperature. The routine clinical chemistry panel, including ALT, AST, and total bile acids, was measured on the Siemens ADVIA 2400 platform using commercially available kits. The Cerner HNA Millennium Laboratory Information System was used to acquire data. Aliquots of serum samples were frozen at -80°C and stored until analysis.

### Sample preparation for LC-MS/MS analysis

Fifty microliter (50 μL) of each serum sample was placed into an individual well of an Ostra protein precipitation plate (Waters Corporation, Milford, MA) on the top of a 96-well collection plate. 400 μL of 1% (v/v) of formic acid in acetonitrile was added to each well for protein precipitation. Deuterated standards of CA, GCA, TCA, CDCA, GCDCA, TCDCA, and DCA were used as internal standards. 10 μL of a 1 μg/mL working solution of internal standard mixture was added to each well of the plate, followed by vortex-mixing. After 10 min of centrifugation, the Ostra plate was removed and supernatant evaporated to dryness under a steady stream of N_2_ at 40°C. Samples were reconstituted in 100 μL of 50% (v/v) methanol in water and 1 μL injected onto the LC/MS system.

### Calibration and quality control standard preparation

Bile acid standard or internal standard (IS) solutions at concentrations of 1 mg/mL were prepared by dissolving each standard in methanol. These individual standard solutions were combined and diluted to achieve the concentration of 20 μg/mL for a working standard solution or 1 μg/mL for a working IS solution. A ten point calibration standard curve (5 ng/mL to 10,000 ng/mL) was prepared by serially diluting the working standard solution into charcoal stripped serum. Quality control (QC) standards were prepared in the same manner (20, 200, and 2000 ng/mL) in charcoal stripped serum. Calibration and QC standards then went through the sample preparation process described above.

### Individual bile acid analysis

IBA analysis was performed by LC/MS/MS. The mass spectrometer was a Sciex (Framingham, MA) 5500 QTrap equipped with Turbo Spray ion source, operating in negative ion mode. This nine bile acid assay was optimized from the previous 3 bile acid method [5] by using a longer column (100 x 2.1mm) at a lower flow rate of 300 μL/min. Mobile phase A was 0.05% (v/v) formic acid in water and mobile phase B was 5% (v/v) acetonitrile in methanol. The gradient program started at 60% B, increased to 90% B in 9 min, decreased to 60% B in 0.1 min, and then held at 60% B for 0.9 min. The mass spectrometer was operated with the source and desolvation temperatures set at 120 and 350°C, respectively. The curtain gas was 40 psi; the ion spray voltage was 4500 V; probe temperature was 600°C; and ion source gas 1 and ion source gas 2 were 40 psi and 30 psi, respectively. Peak integration and quantification was performed using Analyst 1.6.2 software. Individual standard curves for each bile acid were constructed by plotting the ratio of the bile acid peak area to its deuterated standard peak area versus concentration. Slope and y-intercept were calculated using a linear curve fit with 1/x^2^ weighting. The concentrations of bile acids in study samples were calculated relative to the regression line.

Freshly prepared standard curves and QC samples were included in each analysis run. A run was deemed acceptable if the QC samples were ±30% of the nominal concentrations and the coefficient of variance (CV) did not exceed 20%.

### Method assessment

The method performance was assessed using QC samples at three concentration levels (20, 200, and 2000 ng/mL) from the calibration curve. Four replicates of each QC sample were analyzed each day to determine the intra- and inter-day accuracy and precision. This process was repeated three times over three days in order to determine the inter-day accuracy and precision using freshly prepared calibration curves. Accuracy and precision were calculated from the % Relative Error (RE) [% (measured-theoretical)/theoretical concentration] and relative standard deviation [%RSD = % standard deviation/mean], respectively. The assay recovery was evaluated by comparing the mean detector response of extracted QC samples at low, medium, and high concentrations (20, 200, and 1000 ng/mL) in four replicates to those of post-extracted serum blanks spiked at equal concentrations. The matrix effect was estimated by comparing the extracted serum residue and the neat solution.

### Statistical analysis

Data are presented as individuals or group mean ± standard error of the mean (SEM). Statistical analyses were conducted by two-tailed Student t-test to compare the two groups, such as, between healthy groups and patients with liver impairments. Values significantly different are indicated as **p<0.01, and *p<0.05. To examine the predictability of liver injury by the bile acid concentrations, the receiver operating characteristic [6] curve analysis was conducted. The area under ROC curve was used as an overall assessment of a marker’s predictive ability. Principal component analysis (PCA) was conducted on the bile acid concentrations to find a linear combination of the bile acids to account for the maximum variance contained in the original dataset. Hierarchical clustering analysis (HCA) on the bile acid concentrations was conducted to study the correlation between diagnosis groups and the hierarchical clusters. Both PCA and HCA used the transformed data on the logarithmic scale. The analyses were conducted in statistical software packages SAS and JMP.

## Results

### Performance of LC-MS/MS analysis of IBA in human serum

The LC-MS/MS assay developed was able to quantitate a set of 9 IBA: CA, GCA, TCA, CDCA, GCDCA, TCDCA, DCA, GDCA, and TDCA. All IBA had a linear response over a range of 5 to 10,000 ng/mL, with a coefficient of determination (R^2^) above 0.99. The lower quantitation limit for these bile acids was 5 ng/mL. Mean intra-day accuracy was 95–110% for the 9 IBAs, with a mean CV of 1–8%; Mean inter-day accuracy was 91–112%, with a mean CV of 3–13% (S1 Table). The mean recovery for the extraction procedures was 98%-131%. The assay performance was deemed acceptable, and the method was determined to be suitably accurate and precise for these analytes.

### Characterization of bile acids in healthy human subjects

To establish reference ranges for 9 IBA using LC/MS in healthy subjects, serum samples were collected from a total 314 subjects, of which 27 were from healthy subjects participating in clinical trials, 34 healthy volunteers from Pfizer Groton campus, and 253 were from healthy subjects at UM (Table 1). The liver health status of subjects from clinical trials was determined based on physical examination and the status of subjects from UM was determined by medical adjudication based on evaluation of clinical chemistry and medical records.

**Table 1:** Mean serum bile acid concentrations in healthy subjects by age and gender.

Gender	BA^a^	Age <40			Age 40–60			Age >60		
		n = 30			n = 59			n = 28		
		Mean±SEM	10^th^	90^th^	Mean±SEM	10^th^	90^th^	Mean±SEM	10^th^	90^th^
**MALE**	**CA**	48.1 ± 9.9	12.4	108.8	46.0 ±9.5	6.3	82.0	91.0 ± 38.9	6.6	202.6
	**GCA**	192.5 ± 34.7	34.0	509.9	181.3 ± 45.7	32.8	303.2	168.1 ± 39.5	14.4	423.7
	**TCA**	22.3 ± 4.6	4.1	62.2	28.2 ± 7.4	3.3	48.7	29.0 ± 7.5	3.4	90.8
	**CDCA**	196.6 ± 50.9	15.0	469.8	126.7 ± 26.2	14.1	286.6	141.8 ± 53.0	9.5	423.9
	**GCDCA**	631.2 ± 118.6	111.2	1542.0	492.6 ± 103.6	95.3	758.6	431.1 ± 80.3	65.6	1083.0
	**TCDCA**	69.2 ± 11.9	13.5	144.6	81.6 ± 20.9	11.0	146.6	58.2 ± 12.5	12.1	127.6
	**DCA**	196.8 ± 29.6	44.2	316.2	174.9 ± 16.6	30.3	343.0	199.1 ± 39.2	38.8	395.6
	**GDCA**	286.7 ± 69.2	18.3	701.2	211.0 ± 49.0	24.6	364.2	260.1 ± 73.5	23.2	848.6
	**TDCA**	33.1 ± 7.4	1.6	88.6	32.3 ± 7.0	3.4	61.4	37.4 ± 11.1	3.9	99.1
	**TBA**	4.2 ± 0.6	1.5	8.5	4.4 ± 0.9	1.6	6.3	4.0 ± 0.9	1.1	11.8
		Age <40			Age 40–60			Age >60		
		n = 59			n = 98			n = 40		
		Mean±SEM	10^th^	90^th^	Mean±SEM	10^th^	90^th^	Mean±SEM	10^th^	90^th^
**FEMALE**	**CA**	41.3 ± 7.7	6.6	91.5	65.2 ± 15.7	4.2	164.5	103.8 ± 47.3	5.8	160.3
	**GCA**	346.3 ± 172.1	31.2	595.4	209.3 ± 41.7	27.7	418.3	176.8 ± 30.5	24.2	332.9
	**TCA**	78.6 ± 51.3	5.2	77.8	42.2 ± 11.2	3.6	75.4	53.4 ± 23.9	3.5	67.4
	**CDCA**	121.6 ± 23.6	14.2	275.2	122.4 ± 24.4	12.9	299.8	163.0 ± 51.9	13.5	300.8
	**GCDCA**	860.6 ± 303.3	96.3	1326.0	535.0 ± 55.6	94.7	1229.0	500.4 ± 70.5	86.2	1203.0
	**TCDCA**	127.7 ± 44.8	14.4	241.8	80.9 ± 11.4	13.2	161.1	102.0 ± 27.3	13.6	202.2
	**DCA**	207.5 ± 23.3	42.2	442.5	203.3 ± 18.3	40.1	496.8	278.3 ± 50.7	64.9	543.1
	**GDCA**	436.6 ± 134.7	38.1	775.0	315.3 ± 50.0	34.6	612.4	264.2 ± 44.5	55.5	653.2
	**TDCA**	68.4 ± 22.8	6.6	94.1	44.5 ± 7.6	4.5	96.2	59.2 ± 19.4	7.3	106.6
	**TBA**	6.2 ± 1.8	1.2	8.6	4.7 ± 0.5	1.4	8.7	4.7 ± 0.6	1.5	9.5

BA, bile acids; TBA, total bile acids; SEM, standard error of the mean; CA, cholic acid; GCA, glycocholic acid; TCA, taurocholic acid; CDCA, chenodeoxycholic acid; GCDCA, glycochenodeoxycholic acid; TCDCA, taurochenodeoxycholic acid; DCA, deoxycholic acid; TDCA, taurodeoxycholic acid; IBAs, Individual Bile Acids.

^a^ The units for TBA are umol/L; the units for all other BA are ng/mL.

In these healthy subjects, non-conjugated forms of IBA and glycine-conjugated BAs were highly abundant in human serum, with GCDCA found to be the most abundant bile acid (Table 1). The mean concentrations of serum IBA in healthy subjects did not show statistically significant differences among the three age groups (less than 40 years of age, 40–60 years of age and over 60 years of age). The absolute concentrations of the 9 IBA and total bile acids appeared slightly higher in females than in males (S2 Table). The only statistically significant difference observed between genders was the mean DCA concentration in females (p <0.03) as compared to males and more prominently in the 60 and above (p<0.01) group. Upon examination of the overall IBA profiles, which was performed by determining the percentage for each IBA concentration of the sum of all measured IBA, healthy males and females were similar.

**Fig 1:**
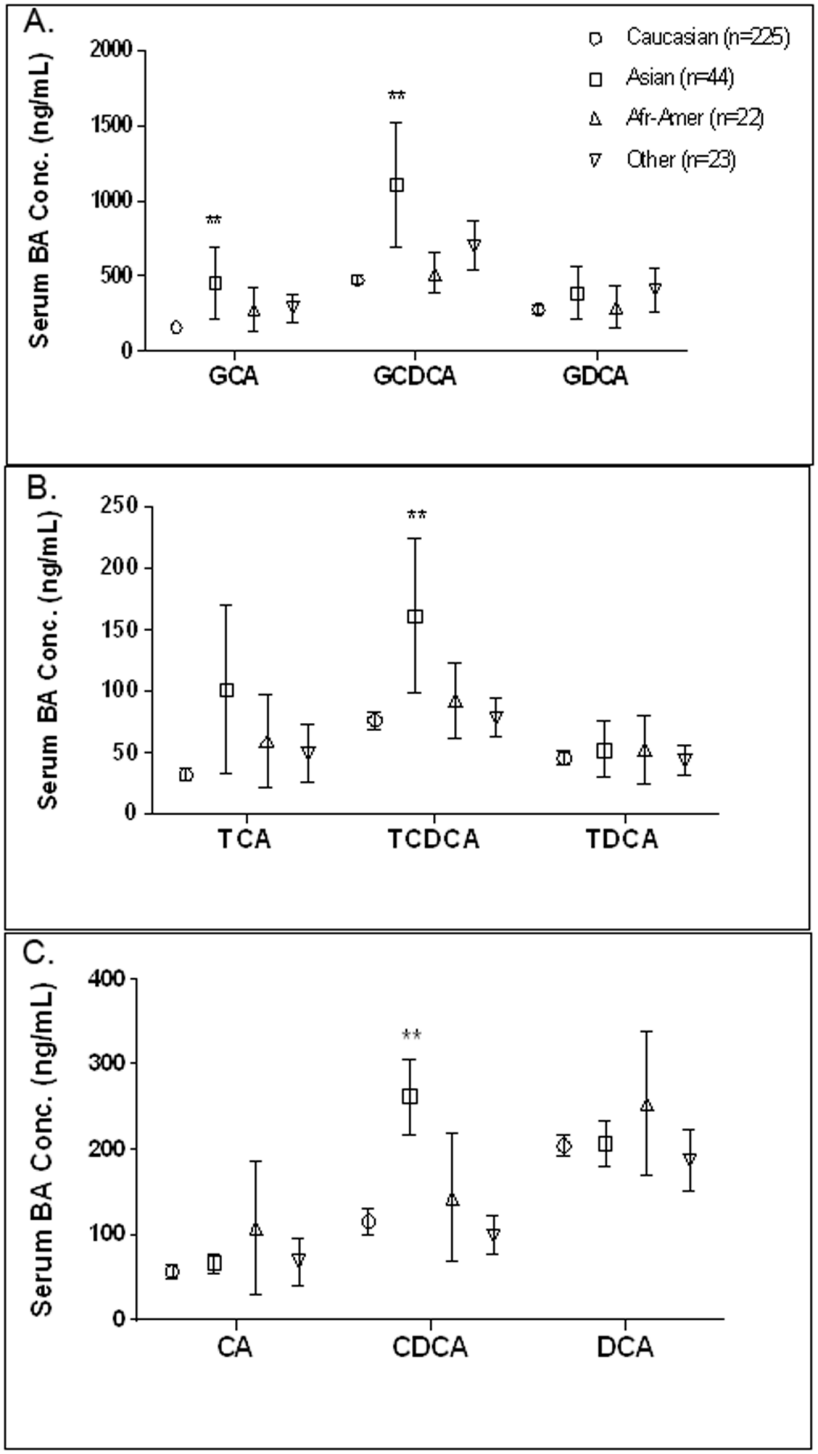
Influence of ethnicity on individual bile acid (IBA) concentrations in healthy subjects. Serum samples from healthy subjects were analyzed for glyco-conjugated bile acids (A), tauro-conjugated bile acids (B), and unconjugated bile acids (C). The values represent average concentrations ± SEM.

When comparing IBA concentrations of healthy subjects across ethnic groups, subjects of Asian origin demonstrated significant differences in four IBA (Fig 1). Although the mean TBA concentration in Asians (6.6±1.9 μmol/L) trended higher than Caucasians (4.2±0.3 μmol/L), the difference was not statistically significant and the mean value was under the generally accepted upper level of normal for healthy subjects (10 μmol/L). However, upon evaluation of IBA, serum concentrations of GCA, GCDCA, CDCA and TCDCA were found to be statistically significantly higher (p<0.01) in Asians as compared to Caucasians.

### IBA in subjects with hepatic impairments

To evaluate the effect of liver injury on serum bile acids we examined 331 samples from patients with a broad range of liver impairments. Overall, as expected, serum TBA concentrations were significantly higher in patients with liver impairments than healthy subjects (p<0.01) (Table 2). When comparing IBA concentrations in healthy subjects and subjects with liver impairments, as expected, the concentrations of IBA were also significantly higher, with the exception of DCA. Furthermore, difference in IBA concentrations between healthy subjects and subjects with liver impairments was larger for conjugated bile acids than for unconjugated bile acids. For instance, CA was 3.1-fold higher in subjects with liver impairments, whereas its taurine conjugated form, TCA showed a 97.2-fold elevation. When comparing serum IBA concentrations across age groups, genders and ethnicity for the liver impaired subjects, no significant difference was observed among the different age groups, as well as for the healthy cohort. However, all 9 IBA were slightly higher in females (n = 145), when compared with males (n = 186) (S1 Fig). Means of IBA in Asians were higher than those in other ethnicities, with slightly larger variability, which may due to the relatively smaller sample size (n = 5). No statistical significance was observed among other ethnicities (S2 Fig).

**Table 2:** Comparison of serum bile acid concentrations between healthy subjects and hepatic injury subjects.

Bile Acid^a^	Healthy	Liver Diseases	DILI (APAP Overdose)	Fold Change		
	Mean ± SEM	Mean ± SEM	Mean ± SEM	DILI/Healthy	DILI/Hepatic	Hepatic/Healthy
**CA**	61.6 ± 9.8	190.7 ± 48.0	889 ± 541	14.4	4.7	3.1
**GCA**	218.3 ± 39.9	6263.8 ± 651.7	11480 ± 4408	52.6	1.8	28.7
**TCA**	41.1 ± 6.0	3995.7 ± 589.4	3270 ± 1264	79.6	0.8	97.2
**CDCA**	115.4 ± 13.5	278.2 ± 50.5	604 ± 341	5.2	2.2	2.4
**GCDCA**	564.5 ± 69.8	7785.7 ± 771.5	16234 ± 5610	28.8	2.1	13.8
**TCDCA**	86.1 ± 11.2	4584.9 ± 448.8	5217 ± 1903	60.6	1.1	53.3
**DCA**	205.6 ± 11.9	88.2 ± 15.5	112 ± 42	0.5	1.3	0.4
**GDCA**	317.0 ± 40.7	997.1 ± 142.5	4096 ± 2099	12.9	4.1	3.1
**TDCA**	48.5 ± 6.3	336.0 ± 45.4	785 ± 356	16.2	2.3	6.9
**TBA**	4.8 ± 0.6	51.3 ± 3.8	110 ± 34	22.9	2.1	10.7

APAP, acetaminophen; BA, bile acid; CA, cholic acid; CDCA, chenodoxycholic acid; DCA, deoxycholic acid; DILI, drug-induced liver injury; TCA, taurocholic acid; GCA, glycocholic acid; GCDCA, glycochenodeoxycholic acid; SEM, standard error of the mean; TCDCA, taurochenodeoxycholic acid; TDCA, tauodeoxycholic acid.

^a^ TBA unit is in umol/L; all other units are ng/mL.

**Fig 2:**
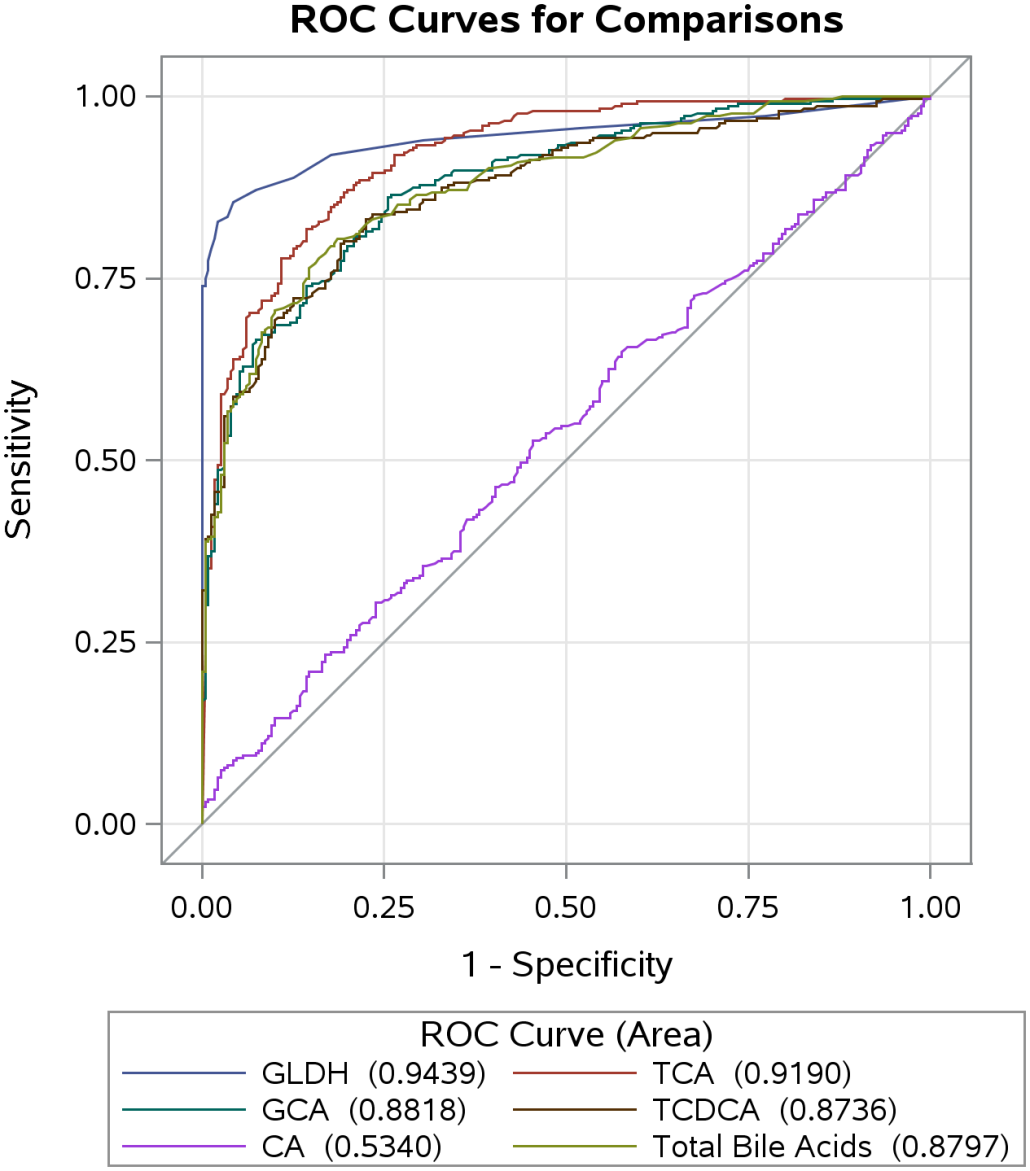
Receiver operating characteristic (ROC) curve for bile acids and liver injury. ROC curve analyses for 331 subjects evaluated the sensitivity and specificity of GLDH, TBA and IBA to detect liver injury. Liver injury was defined using a biochemical criterion [15] (b-id = "pxrfzr0 2x ALP or ≥3x ALT and ≥ 2x TBil). The values represented AUC for individual parameters.

To assess the potential of IBA to differentiate subjects with liver impairments from healthy subjects, we performed a receiver operating characteristic curve (ROC) analysis. This analysis evaluates the sensitivity and specificity of each IBA to detect liver injury (Fig 2). To define liver injury we used a clinical chemistry based criterion developed by an international working group for DILI [15]. This criterion consists of Hy’s Law (≥3x ALT and ≥2x TBil), which is used to identify severe cases of liver injury; the hepatocellular damage is detected by increases of alanine aminotransferase (≥5x ALT) and hepatobiliary damage by increases of alkaline phosphatase (≥2xALP). TCA had the highest diagnostic power with an area under the curve of 0.919, followed by GCA (AUC 0.882), total BAs (AUC 0.880), and TCDCA (AUC 0.874). CA exhibited the least diagnostic power with an AUC of 0.534.

**Fig 3:**
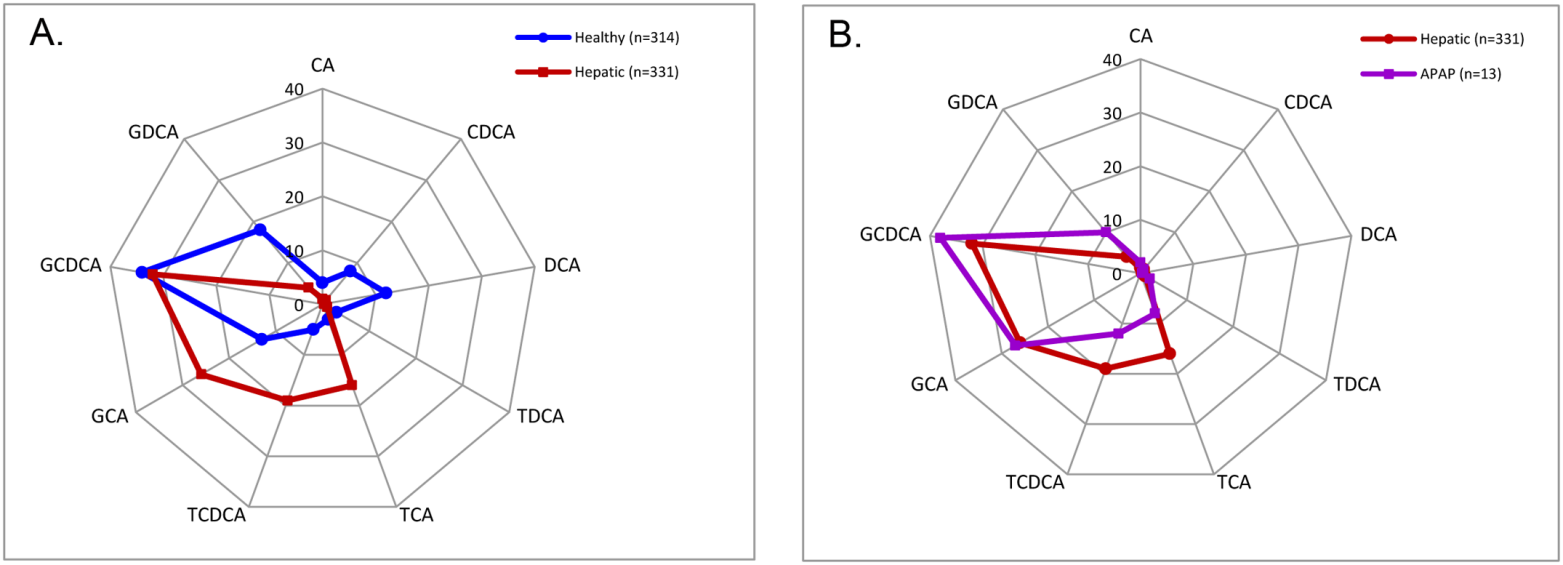
Serum bile acid composition changes among healthy, hepatic injury, and drug-induced liver injury (DILI) subjects. (A) Bile acid pattern comparisons between healthy and hepatic injury subjects demonstrated differences between the two groups. (B) Slight differences in the bile acid patterns of APAP overdosed patients (DILI) and all hepatic injury subjects were observed. Numbers were expressed as a percent of the total individual bile acids measured.

To assess whether IBA analysis might provide additional advantage in detection of liver injury in humans, the profiles of measured IBA in serum of healthy subjects and subjects with liver impairments were compared (Fig 3). As outlined previously, profiles were determined using the percentage for each IBA concentration of the sum of all IBA measured. In comparison to healthy subjects, subjects with hepatic impairments exhibited distinct bile acid profiles (Fig 3). TCA accounted for 3% of the 9 bile acids measured in healthy volunteers, yet in patients with liver impairments TCA accounted for 16% of the measured bile acid species. On the other hand, DCA accounted for 12% of the 9 measured bile acids in the healthy population, while only 0.3% in the patients with liver impairments. Interestingly, the relative proportions of GCDCA, the most abundant bile acid species in human serum, were indistinguishable between the healthy and hepatic impairment populations (34% versus 32%). Overall, a greater percentage of CA and its conjugates (TCA and GCA) was observed in healthy subjects (43%) than subjects with liver impairments (20%). In contrast, a lower percentage of secondary bile acids (DCA, GDCA, and TDCA) was found in healthy subjects (33%) than in subjects with liver impairments (5%). Four of the conjugated bile acids (GCA, GCDCA, TCA, and TCDCA) accounted for 93% of the 9 bile acids in subjects with liver impairments, while in healthy subjects they accounted only for 55%. Since the cohort of subjects with liver impairments consisted of mainly subjects with liver diseases, we examined whether the IBA profile was similar for cases of acute drug induced liver injury. When comparing APAP overdosed patients with all other hepatic injury subjects, only slight differences were observed in the IBA profiles of APAP overdosed subjects, including a slightly higher proportion of glyco-conjugates and slightly smaller proportion of tauro-conjugates (Fig 3).

### Bile acid patterns associated with different liver impairments

**Fig 4:**
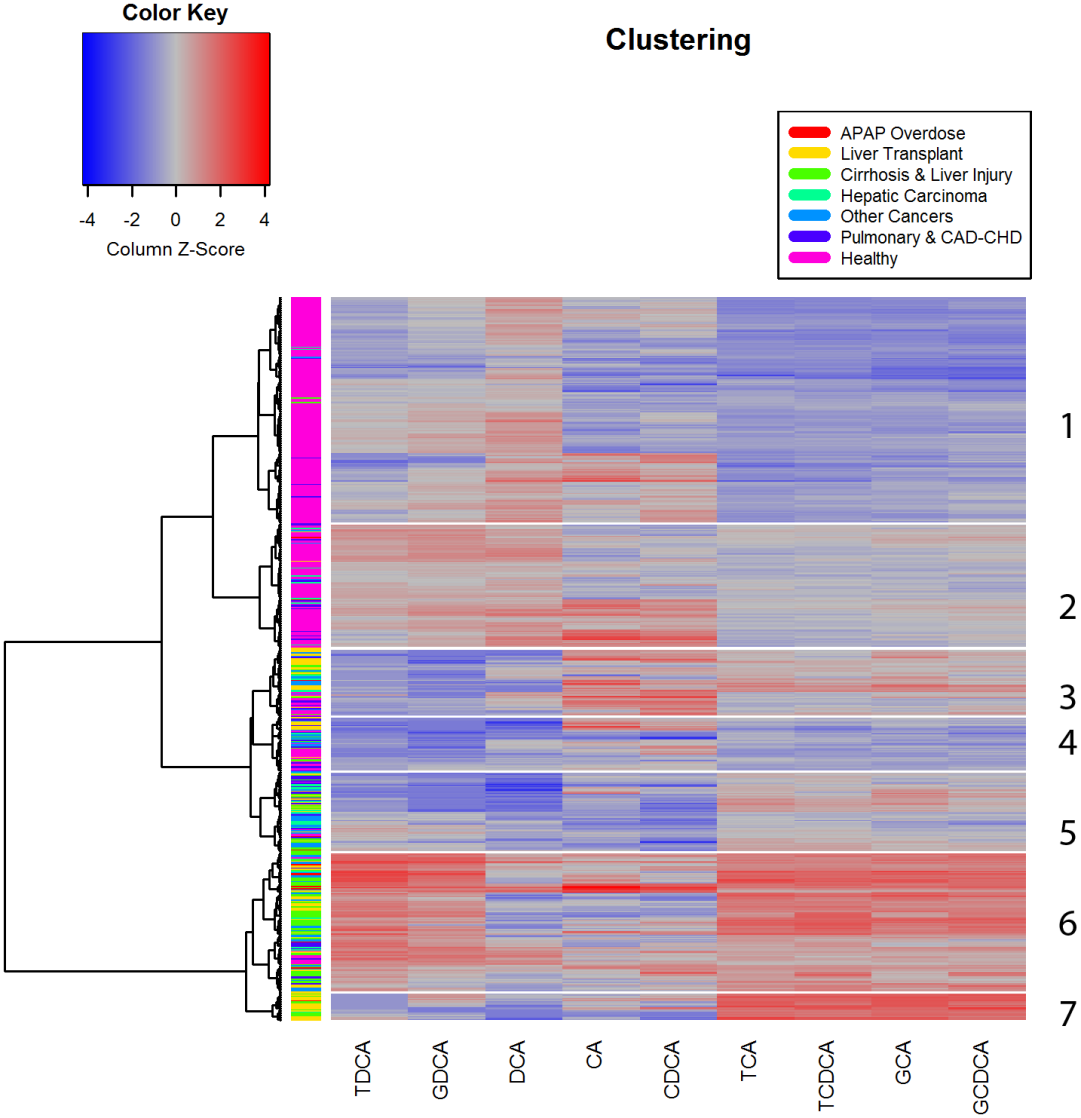
Hierarchical clustering and heat map analysis of 9 individual bile acids. Cluster analysis differentiated a total of 7 clusters, and showed distinct bile acid patterns across different diagnosis groups.

To examine whether IBA analysis could differentiate subjects based on the etiology of liver impairments and/or provide mechanistic insights, we evaluated the patterns of serum bile acids in healthy subjects vs subjects of different impairments using cluster analysis (Fig 4). Subjects with liver impairments were assigned to groups based on primary diagnosis (a) liver transplant within the last 3 years, (b) hepatic carcinoma (diagnosed by biopsy or resection), (c) coronary artery disease (CAD)- coronary heart disease (CHD), cirrhosis and liver injury (Hepatitis B or C, hepatic graft vs. host disease, ethanol cirrhosis, *etc*), pulmonary (Influenza A, H1N1 Influenza, acute respiratory distress syndrome), and acetaminophen toxicity (APAP-induced liver failure).

As shown in Fig 4, Tables 3 and 4, Clusters 1 and 2 contained 310 subjects and featured relatively low concentrations of TCA, GCA, TCDCA, and GCDCA. Clusters 1 and 2 included predominantly healthy subjects (87%). In contrast, clusters 6 and 7, comprising of 114 subjects, featured relatively high concentrations of TCA, GCA, TCDCA, and GCDCA and included the majority of subjects with cirrhosis (67.1%), APAP overdose (61.6%), and a substantial portion of subjects with liver transplant (48.1%). The high values of DCA and its conjugates (TDCA and GDCA) in cluster 4 were associated with subjects diagnosed with cardiac and heart diseases along with liver impairments.

**Table 3:** Distribution of subjects by diagnostic groups.

Cluster	Diagnostic Groups						
	Healthy % (N)^a^	APAP Overdose % (N)	Liver Transplant % (N)	Cirrhosis & Liver Injury % (N)	Hepatic Carcinoma % (N)	Other Cancers % (N)	Pulmonary & CAD-CHD % (N)
1	60.8 (191)	0 (0)	0 (0)	1.0 (2)	1.0 (2)	0.5 (1)	2.0 (4)
2	26.8 (84)	15.3 (2)	1.8 (2)	0 (0)	6.4 (7)	2.7 (3)	10.9 (12)
3	3.5 (11)	7.7 (1)	25.9 (21)	8.9 (7)	12.1 (4)	17.5 (11)	8.9 (5)
4	4.8 (15)	0 (0)	9.9 (8)	6.3 (5)	6.1 (2)	12.7 (8)	19.6 (11)
5	1.3 (4)	15.4 (2)	13.6 (11)	15.2 (12)	36.4 (12)	25.4 (16)	25.0 (14)
6	2.9 (9)	53.9 (7)	29.6 (24)	57.0 (45)	15.25 (5)	38.1 (24)	17.9 (10)
7	0 (0)	7.7 (1)	18.5 (15)	10.1 (8)	3.0 (1)	0 (0)	0 (0)
Total	100 (314)	100 (13)	100 (81)	100 (79)	100 (33)	100 (63)	100 (56)

^a^ % refers to % of subjects in the diagnosis group; N refers to the number of subjects in the cluster

**Table 4:** Distribution of subjects by hierarchical clusters.

Diagnosis	Hierarchical Cluster Number						
	1 % (N)^a^	2 % (N)	3 % (N)	4 % (N)	5 % (N)	6 % (N)	7 % (N)
Healthy	95.5 (191)	76.4 (84)	18.3 (11)	30.6 (15)	5.6 (4)	7.3 (9)	0 (0)
APAP Overdose	0 (0)	1.8 (2)	1.7 (1)	0 (0)	2.8 (2)	5.7 (7)	4.0 (1)
Liver Transplant	0 (0)	1.8 (2)	35.0 (21)	16.3 (8)	15.5 (11)	19.4 (24)	60.0 (15)
Cirrhosis & Liver Injury	1.0 (2)	0 (0)	11.7 (7)	10.2 (5)	16.9 (12)	36.3 (45)	32.0 (8)
Hepatic Carcinoma	1.0 (2)	6.4 (7)	6.7 (4)	4.1 (2)	16.9 (12)	4.0 (5)	4.0 (1)
Other Cancers	0.5 (1)	2.7 (3)	18.3 (11)	16.3 (8)	22.5 (16)	19.4 (24)	0 (0)
Pulmonary & CAD-CHD	2.0 (4)	10.9 (12)	8.3 (5)	22.5 (11)	19.7 (14)	8.1 (10)	0 (0)
Total	100 (200)	100 (110)	100 (60)	100 (49)	100 (71)	100 (124)	100 (25)

^a^ % refers to % of subjects in the cluster; N refers to the number of subjects in the cluster.

## Discussion

In this study, we utilized a LC-MS/MS assay to measure nine bile acids in a large cohort of healthy human subjects and subjects with liver impairments to evaluate their potential as biomarkers. Since BA synthesis, metabolism and excretion are affected by liver diseases, IBAs and their profiles have been studied as both diagnostic and prognostic markers [16]. Even through there are many bile acid species in humans, CA, CDCA, DCA and their glycine and taurine conjugates represent 95%-99% of the bile acid pool in systemic circulation upon liver injury, and are the only bile acids that accumulate to micro molar concentrations in serum [8, 17, 18]. Therefore, the focus of this investigation was on these nine bile acids. Our objective was to evaluate the performance of IBA as biomarkers of liver injury in a real world setting, thus the pre-analytical (sample collection) requirements for sampling was minimal, so, for example, fasting was not necessary. Compared with previous publications that utilized more strict controls, such as limited food and alcohol intake, the data reported in this study represents a real-world approach, utilizing subjects with significant life events (impaired liver function), thus providing insight into the robustness of IBAs as potential biomarkers for liver injury and diseases. The Clinical and Laboratory Standards Institute (CLSI) recommends using at least 120 samples for establishing reference ranges in healthy individuals (ref document C28 A3). The samples analyzed consisted of 314 total samples from healthy human subjects, the largest population reported to date. In general, the mean concentrations of each IBA in this study were higher than those reported from fasted, healthy subjects [19–23]. For example, the mean value of the most abundant bile acid in humans, serum GCDCA, was reported as 745 ± 54.2 nM from 150 fasted healthy volunteers [23], whereas in our study was determined it to be 1,297 ± 144 nM including both fasted and non-fasted individuals. This difference observed is most likely due to the fact that bile acids are typically elevated in non-fasted individuals [21]. However, Scherer *et al* [24] reported serum GCDCA levels of 1,710 nM from 29 fasted healthy subjects, suggesting that the differences observed could be a reflection of biological variance or assay platform variability. Bathena *et al*. reported that factors, such as food and drink, had significant impact on the absolute bile acid concentrations, yet the relative proportions of bile acid species were only slightly affected [19]. The Mayo Clinic reported reference values for several bile acids with their LC/MS-MS method [25]. In the Mayo study, total cholic acid concentrations (CA+TCA+GCA) and total chenodeoxycholic acids concentrations (CDCA +TCDCA+GCDCA) were ≤5μM and ≤6μM, respectively. Those findings were in line with our data, which were 0.7μM and 1.8μM, respectively. Considering differences in analytical methodologies and pre-analytical conditions, the IBAs concentrations observed in our study were in line overall with other IBA analyses in healthy human subjects.

To further characterize IBA in healthy human subjects, we evaluated the impact of gender, age, and ethnicity on the serum IBA concentrations. We did not find significant differences in IBA concentrations among age groups, which was consistent with previous studies for both individual and total bile acids [26–29]. Furthermore, in work by *Bathena et al*, no significant effect of age was seen on either serum or the urinary BA profiles by using BA indices that described composition, sulfation and amidation of total and individual BAs, instead of absolute BA concentrations [30]. The data assessment for any gender effect on IBA concentrations was consistent with previous studies by *Kawasaki 1986 and Praneeth 2014*, suggesting that no significant sex-related differences exist for serum bile acids in all age groups for humans [19, 31]. However, our study demonstrated significant differences in IBA concentration among ethnic groups, with subjects of Asian descent displaying significantly higher concentrations of CDCA, TCDCA, GCDCA and GCA than subjects of other ethnic backgrounds. To our best knowledge, this is the first report demonstrating ethnic differences in serum IBAs. Costarelli *et al*. reported fecal concentrations of TBA were significantly higher in Asian vegetarians than Caucasian vegetarians [32]. These different bile acid concentrations may be due to genetic differences in bile acid synthesis/metabolism or possibly different dietary patterns. This difference may also be related to the fact that Asians are more susceptible to certain liver diseases. Further research is needed to understand these differences and their significance.

Increases observed for both total and some individual bile acid concentrations in subjects with liver impairments as compared with healthy subjects were consistent with other reports [17, 33, 34]. Trottier reported an increased ratio of tauro to glyco-conjugates in patients with primary biliary cirrhosis [8]. Our results also demonstrated that the ratio of tauro-conjugates to glyco-conjugates was increased in the hepatic injury patients (from 0.16 to 0.59, Table 2) suggesting that tauro-conjugates could be relatively more sensitive markers in liver injury. This is supported by the ROC analysis indicating that TCA had the strongest diagnostic power of all the bile acids analyzed here in Fig 2 [35].

In this work, significantly lower concentrations of DCA were observed in subjects with liver impairments when compared with healthy subjects. Recently, DCA was reported as significantly reduced in patients with biliary tract disease [16]. Similar results reported in other studies, where a marked increase in the proportions of primary BAs and a decrease in the proportions of secondary BA, such as DCA, were observed in patients with cholestatic liver diseases [8, 23, 34, 36]. This may imply that there is a significant impairment of dehydroxylation of secondary BAs in patients with primary liver diseases, since secondary BAs are formed through an enzymatic dihydroxylation of primary BAs catalyzed by bacterial enzymes in the intestine. Lake *et al*. also reported that there were elevated taurine-conjugated BAs and decreased concentrations of CA and GDCA in the livers from patients with non-alcoholic steatohepatitis, and revealed that a potential shift toward the alternative pathway of BA synthesis based on a transcriptomic analysis of 70 BAs [37]. The unconjugated BA, CA, CDCA, and DCA, are more hydrophobic, and thus more cytotoxic, with DCA being one of the most toxic/cytotoxic species. Accumulation of these hydrophobic BAs could result in mitochondrial damage, disruption of cell membranes and production of ROS in hepatocytes, ultimately leading to apoptosis and necrosis [38–41]. Changes in BA composition are known to lead to altered hydrophobicity of total BAs, which subsequently yield a decrease in the hydrophobicity index in liver injury patients. A reduction of hydrophobicity suggests that these patients may present a less toxic BA pool, when compared to healthy subjects. While the predominant hypothesis in the field of cholestatic liver injury is that the accumulation of bile acids in serum and liver directly causes cell death, recent work has cast some doubt on this hypothesis [42]. Our observations were in line with previous reports in that the unconjugated BA pool did not become elevated [23, 34, 43, 44]. These data support the premise that changes in IBAs are an indicator of liver injury and dysfunction, but not a direct cause of cell death.

In our study, subjects with accidental overdoses of APAP provided the only cases of drug induced liver injury known in the sample pool. Interestingly, these subjects had higher mean serum concentrations of the unconjugated bile acids CA and CDCA as compared with healthy subjects and the entire population of subjects with liver impairment (Table 2). This observation was consistent with our findings in a rodent model that demonstrated a significant increase in serum levels of CA in conjunction with APAP-induced hepatocyte damage [5], indicating the potential for translatability from animal species to humans. Significant increases were also observed for GCA and TCA in rats administered APAP when compared to controls; the mean values for these BA were also observed to be higher in APAP overdose subjects as compared with healthy subjects, further demonstrating the potential for translation. GDCA concentration in DILI group was also much higher than other liver diseases. Woolbright *et al*. recently reported that GDCA was significantly increased in APAP induced, non-surviving acute liver failure and suggested that GDCA may serve as a prognostic biomarker [18]. This point is supported by our data, even with a small population size (n = 13).

Most researchers have looked at individual bile acids from the perspective of individual concentrations or patterns. We have illustrated that even though significant differences can be observed in Asians when compared to Caucasians, the overall composition profile in healthy Asians matches that of healthy Caucasians. The composition profiles may be the key to teasing out the questions regarding individual bile acids as biomarkers. The composition profile of the liver impaired group did look different from healthy individuals, demonstrating that significant compositional changes were related to liver impairment and not ethnicity. This method of data illustration may provide a more powerful analysis going forward for investigations of bile acids as biomarkers.

One of the main goals of this study was to investigate the relationship between the etiologies of liver diseases and BA patterns. In our study, the concentrations of BAs conjugated with taurine and glycine were elevated in patients with different types of liver diseases compared to healthy volunteers. Thus, elevation of the concentrations of taurine- and glycine conjugates may not be specific to a single type of liver injury. However, cluster analysis revealed that the high values of conjugated primary BAs, including TCA, GCA, TCDCA, and GCDCA, were associated with primary liver diseases, such as APAP overdose, liver transplant and chronic liver injury. The sustained increase in these conjugated BAs suggested that specific BAs may serve as novel biomarkers for liver injury or liver dysfunction that could differentiate a variety of liver injury etiologies. On the other hand, the high concentrations of secondary bile acid DCA and its conjugates were associated with the clusters of subjects with liver impairment that also had been diagnosed with cardiovascular diseases. BA are known to play a role in modulating cardiovascular function [45] and these patterns may provide insight in these roles. Therefore, these data provide evidence that different BA patterns are linked to various diseases and have the potential to serve as biomarkers. Further work is needed to understand this complicated relationship.

## Conclusions

This study provided normal serum reference ranges for 9 individual bile acids in a large population of human subjects with no consideration to diet, alcohol, smoking, or other factors. Significant differences for bile acid concentrations were demonstrated for subjects of Asian ethnicity and differences were observed for subjects with liver impairment. We showed that defining normal serum concentration ranges alone for individual bile acids may not be as useful as defining normal profiles to remove confounding factors, such as ethnicity. This data demonstrated the ability to separate out disease populations based on their bile acid patterns. The utility of combining the bile acid profiles with the analysis of bile acid patterns has the potential for differential diagnosis of liver diseases and provide a better path forward for the evaluation of individual bile acids as biomarkers.

## Supporting information

S1 Table Performance of the quality control standards in the LC/MS/MS quantitative assay for nine IBA. (DOCX)

S2 Table Reference ranges for serum IBAs in healthy populations. (DOCX)

S3 Table General information for healthy subjects. (XLSX)

S4 Table General information for subjects with hepatic injury. (XLSX)

S1 Fig IBA in liver impaired subjects. (TIF)

S2 Fig Influence of ethnicity in liver impaired subjects. (TIF)

S3 Fig Principal component analysis: Three groups of bile acids. (TIF)
