# Supplementary material for: Assessment of serum bile acid profiles as biomarkers of liver injury and liver disease in humans
Source: PLoS One. 2018 Mar 7;13(3):e0193824. doi: 10.1371/journal.pone.0193824 (PMC5841799; doi:10.1371/journal.pone.0193824)
Supplement: S1 Table — (DOCX) [file pone.0193824.s001.docx]

**S1 Table. Performance of the quality control standards in the LC/MS/MS quantitative assay for nine IBA**

| IBA | |  | 0.02 µg/mL | | |  | 0.2 µg/mL | | |  | 2 µg/mL | | |
| --- | --- | --- | --- | --- | --- | --- | --- | --- | --- | --- | --- | --- | --- |
|  | |  | Avg (µg/mL) | RSD% | RE% |  | Avg (µg/mL) | RSD% | RE% |  | Avg (µg/mL) | RSD% | RE% |
| Intra-day validation (n=4) | | | |  |  |  |  |  |  |  |  |  |  |
| CA |  | | 0.0211 | 1 | 105 |  | 0.206 | 4 | 103 |  | 1.99 | 2 | 100 |
| CDCA |  | | 0.0199 | 4 | 99 |  | 0.204 | 3 | 102 |  | 1.90 | 2 | 95 |
| DCA |  | | 0.0209 | 3 | 104 |  | 0.220 | 4 | 110 |  | 2.00 | 2 | 100 |
| GCA |  | | 0.0200 | 5 | 100 |  | 0.208 | 5 | 104 |  | 2.06 | 4 | 103 |
| GCDCA |  | | 0.0211 | 3 | 105 |  | 0.208 | 5 | 104 |  | 2.08 | 4 | 104 |
| GDCA |  | | 0.0200 | 2 | 100 |  | 0.202 | 1 | 101 |  | 2.04 | 3 | 102 |
| TCA |  | | 0.0212 | 2 | 106 |  | 0.218 | 7 | 109 |  | 1.97 | 5 | 99 |
| TCDCA |  | | 0.0203 | 4 | 102 |  | 0.209 | 7 | 104 |  | 1.92 | 4 | 96 |
| TDCA |  | | 0.0196 | 5 | 98 |  | 0.205 | 8 | 102 |  | 1.97 | 3 | 98 |
|  |  | |  |  |  |  |  |  |  |  |  |  |  |
| Inter-day validation (n=4) | | | |  |  |  |  |  |  |  |  |  |  |
| CA |  | | 0.0212 | 3 | 106 |  | 0.217 | 8 | 109 |  | 1.89 | 11 | 94 |
| CDCA |  | | 0.0211 | 6 | 105 |  | 0.219 | 7 | 110 |  | 1.95 | 13 | 98 |
| DCA |  | | 0.0217 | 5 | 108 |  | 0.223 | 5 | 112 |  | 1.99 | 4 | 100 |
| GCA |  | | 0.0211 | 7 | 105 |  | 0.219 | 8 | 109 |  | 1.97 | 9 | 99 |
| GCDCA |  | | 0.0212 | 6 | 106 |  | 0.222 | 7 | 111 |  | 1.94 | 9 | 97 |
| GDCA |  | | 0.0201 | 4 | 101 |  | 0.213 | 6 | 107 |  | 2.16 | 5 | 108 |
| TCA |  | | 0.0206 | 5 | 103 |  | 0.209 | 10 | 105 |  | 1.92 | 11 | 96 |
| TCDCA |  | | 0.0205 | 5 | 103 |  | 0.215 | 8 | 108 |  | 1.85 | 7 | 92 |
| TDCA |  | | 0.0204 | 10 | 102 |  | 0.213 | 10 | 106 |  | 1.83 | 11 | 91 |
| CA, cholic acid ; GCA, glycocholic acid ; TCA, taurocholic acid; CDCA, chenodeoxycholic acid; GCDCA, glycochenodeoxycholic acid; GDCA, glycodeoxycholic acid; TCDCA, taurochenodeoxycholic acid; DCA, deoxycholic acid; TDCA, taurodeoxycholic acid; | | | | | | | | | | | | | |
| IBA, Individual Bile Acids; %RSD= % relative standard deviation; %RE=% relative error | | | | | | | | | | | | | |
|  | | | | | | | | | | | | | |
