# Supplementary material for: Assessment of serum bile acid profiles as biomarkers of liver injury and liver disease in humans
Source: PLoS One. 2018 Mar 7;13(3):e0193824. doi: 10.1371/journal.pone.0193824 (PMC5841799; doi:10.1371/journal.pone.0193824)
Supplement: S2 Table — (DOCX) [file pone.0193824.s002.docx]

**S2 Table. Reference ranges for serum IBA in healthy populations**

|  | Overall Mean±SEM | 10^th^ | 90^th^ | Female Mean±SEM | 10^th^ | 90^th^ | Male Mean±SEM | 10^th^ | 90^th^ |
| --- | --- | --- | --- | --- | --- | --- | --- | --- | --- |
| BA | n=314 | | | n=197 | | | n=117 | | |
| TCA | 44.8±10.8 | 3.6 | 67.3 | 55.5±17.0 | 3.7 | 73.4 | 26.9±4.3 | 3.4 | 64.5 |
| CA | 62.7±8.8 | 5.8 | 143.2 | 65.9±12.6 | 5.4 | 155.2 | 57.4±10.8 | 8.1 | 121.0 |
| GCA | 220.3±36.3 | 27.7 | 429.7 | 243.7±55.6 | 28.2 | 429.4 | 181.0±26.3 | 26.5 | 429.4 |
| CDCA | 136.9±13.8 | 12.3 | 337.0 | 130.5±17.5 | 13.3 | 288.0 | 147.8±22.4 | 12.0 | 388.0 |
| GCDCA | 583.7±64.7 | 78.5 | 1197.0 | 625.5±95.9 | 87.8 | 1262.0 | 513.4±63.4 | 73.7 | 1126.0 |
| TCDCA | 89.3±10.6 | 13.2 | 180.3 | 99.2±15.5 | 13.3 | 201.4 | 72.8±11.3 | 12.9 | 142.4 |
| TDCA | 46.8±5.8 | 4.1 | 92.8 | 54.6±8.7 | 5.3 | 99.3 | 33.7±4.8 | 2.9 | 83.6 |
| DCA | 207.4±11.1 | 40.6 | 438.9 | 219.9±15.4 | 43.9 | 492.5 | 186.2±14.6 | 32.0 | 354.0 |
| GDCA | 303.8±33.0 | 29.1 | 652.6 | 340.4±47.9 | 38.7 | 735.5 | 239.8±35.0 | 21.3 | 497.0 |
| TBA | 4.8±0.4 | 1.2 | 8.9 | 5.1±0.6 | 1.2 | 9.4 | 4.3±0.5 | 1.2 | 8.6 |
| BA, Bile Acids; TBA, total bile acids; SEM, standard error of the mean; CA, cholic acid; GCA, glycocholic acid; TCA, taurocholic acid; CDCA, chenodeoxycholic acid; GCDCA, glycochenodeoxycholic acid; TCDCA, taurochenodeoxycholic acid; DCA, deoxycholic acid; TDCA, taurodeoxycholic acid. | | | | | | | | | |
| The unit for IBA is ng/mL; the unit for TBA is umol/L. | | | | | | | | | |
|  | | | | | | | | | |
