# Supplementary figures and images for: Assessment of serum bile acid profiles as biomarkers of liver injury and liver disease in humans
Source: PLoS One. 2018 Mar 7;13(3):e0193824. doi: 10.1371/journal.pone.0193824 (PMC5841799; doi:10.1371/journal.pone.0193824)

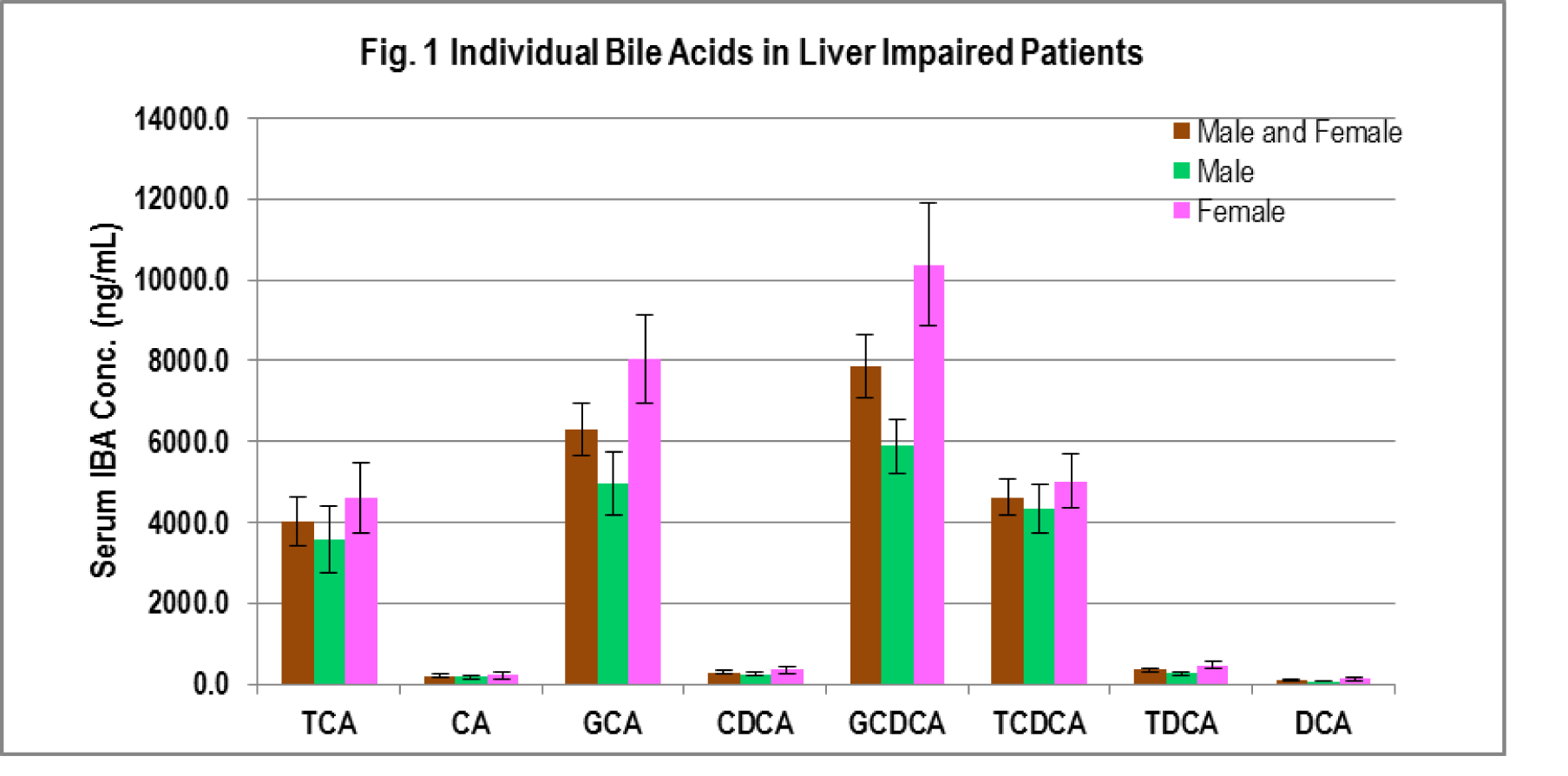

Supplement: S1 Fig — (TIF) [file pone.0193824.s005.tif]

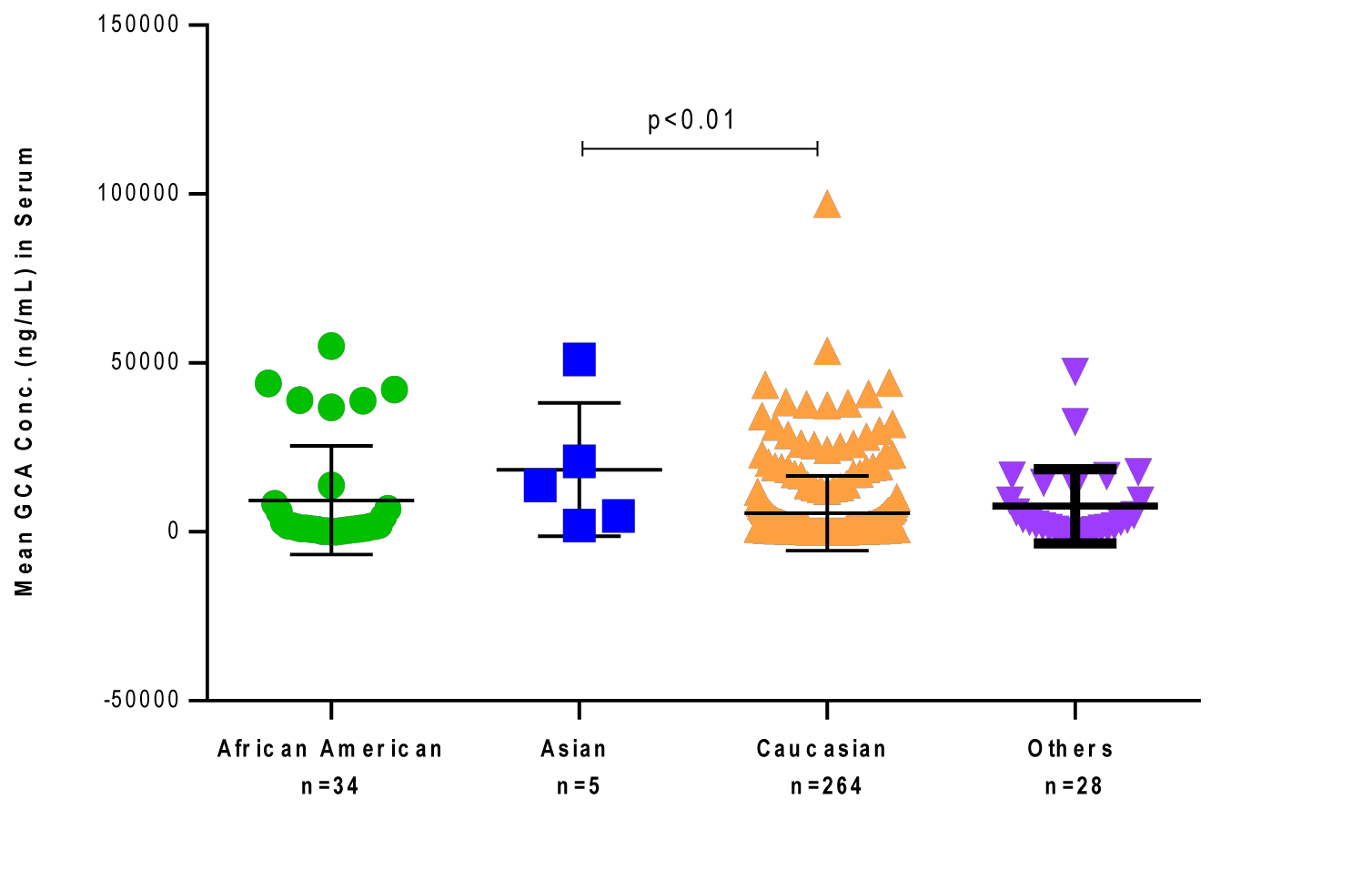

Supplement: S2 Fig — (TIF) [file pone.0193824.s006.tif]

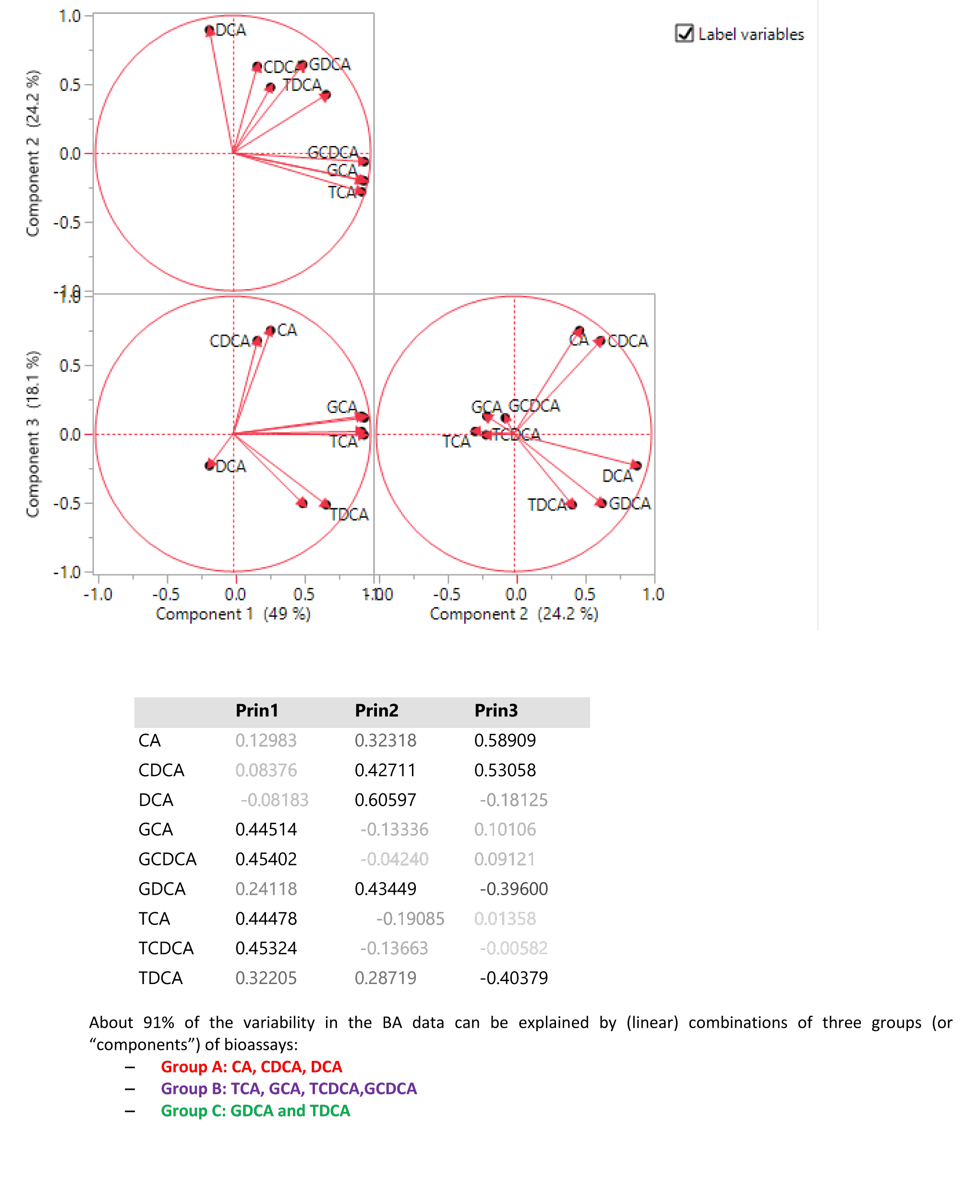

Supplement: S3 Fig — (TIF) [file pone.0193824.s007.tif]
